# Estradiol Promotes Myelin Repair in the Spinal Cord of Female Mice in a CXCR4 Chemokine Receptor-Independent Manner

**DOI:** 10.3390/ijms26104752

**Published:** 2025-05-15

**Authors:** Marianne Bardy-Lagarde, Narimene Asbelaoui, Michael Schumacher, Abdel Mouman Ghoumari

**Affiliations:** UMR1195, Inserm and University Paris-Saclay, 80, Rue du Général Leclerc, 94276 Kremlin-Bicêtre, France

**Keywords:** myelin, estradiol, testosterone, CXCR4, astrocytes, oligodendrocytes, Schwann cells

## Abstract

In the adult central nervous system (CNS), myelin regeneration primarily occurs through the differentiation of oligodendrocyte progenitor cells into mature oligodendrocytes. In men, declining testosterone levels accelerate the progression of multiple sclerosis (MS), while in women, menopause worsens MS-related disability. We previously demonstrated that functional testes and testosterone are required for the spontaneous remyelination of a focal lysolecithin (LPC)-induced demyelinating lesion in the spinal cords of male mice. Testosterone-dependent myelin repair was dependent on the induction of the chemokine receptor CXCR4 in astrocytes that repopulated the lesion and on cooperation between androgen-receptor signaling and CXCR4 signaling. In the present study, we investigated whether ovaries and estradiol have a comparable key role in female mice. Ovariectomy prevents, the appearance of astrocytes, while treatment with estradiol enhances astrocyte numbers and promotes remyelination by oligodendrocytes within the LPC-demyelinated lesion. Unlike testosterone, estradiol did not induce CXCR4 expression, and its effects remained unaffected by the CXCR4 inhibitor AMD3100. As was seen with testosterone treatment, the presence of astrocytes and myelinating oligodendrocytes within the LPC lesion of estradiol-treated females prevented the incursion of Schwann cells. These findings highlight estradiol’s crucial role in CNS remyelination in females, providing a strong rationale for estrogen-replacement therapy in estrogen-deficient and menopausal women with MS.

## 1. Introduction

In the vertebrate central nervous system (CNS), the insulating myelin sheaths surrounding axons are formed by myelinating oligodendrocytes (OLs). They facilitate the rapid transmission of action potentials and play a critical role in the maturation of excitatory domains on axons, potassium buffering, and metabolic support via lactate—a key substrate for neuronal energy metabolism. Disruptions in these functions, as observed in traumatic brain injuries, Alzheimer’s disease, and multiple sclerosis, contribute to axonal dysfunction and neurodegeneration, underscoring the therapeutic potential of targeting oligodendrocyte–neuron interactions for neuroprotection and repair [1,2,3]. Myelin is damaged or lost in demyelinating diseases such as multiple sclerosis (MS) and also in response to ischemic or traumatic brain injury, as oligodendrocytes are particularly vulnerable cells because of their high energy requirements and sensitivity to glutamate toxicity [4,5,6].

In the adult brain and spinal cord, damaged myelin and lost oligodendrocytes can be replaced by a process known as remyelination or myelin repair, which involves oligodendrocyte progenitor cells (OPCs). These stem-like cells are widely distributed in the adult rodent brain and spinal cord [7]. However, despite relatively stable densities of parenchymal OPCs, myelin repair declines with age and MS progression, often failing to occur in response to injury. One reason seems to be the loss of the capacity of OPCs to differentiate into myelinating oligodendrocytes [5,6,7,8]. Enhancing the regenerative capacity of myelin and promoting OPC maturation have therefore become key targets in the development of therapeutic strategies promoting myelin repair.

The ability of oligodendrocytes to repair myelin depends on their environment, with several identified molecules, such as growth factors, the Nogo receptor, the canonical Wnt pathway, and the nuclear receptor retinoid X receptor-γ (RXRγ) having been shown to stimulate remyelination by oligodendrocytes [9]. However, an often-neglected aspect of remyelinating therapies is the hormonal status of patients. Thus, low levels of testosterone and testicular hypofunction have been associated with worse clinical outcomes in men with MS [10,11]. Using an experimental model of lysolecithin (LPC)-induced demyelination and spontaneous remyelination of the spinal white matter in male mice, we demonstrated key roles for testosterone and the androgen receptor (AR) in myelin repair. Indeed, the regeneration of myelin by oligodendrocytes was compromised after castration of mice and restored by treatment with male-like levels of testosterone (10–15 nM). Moreover, the conditional ablation of AR in the CNS, or selectively within astrocytes, abolished testosterone-dependent remyelination [12,13]. Testosterone treatment of male mice also reduced neurological disability in the experimental autoimmune encephalomyelitis (EAE) model of MS [14,15].

Our recent work has provided insight into a mechanism of testosterone-dependent myelin repair in the spinal cords of male mice. We showed that astrocytes appearing in the area of LPC-induced demyelination express AR, CXCR4, and its endogenous chemokine ligand, CXCL12. We also demonstrated that testosterone and CXCR4 stimulate in an interdependent manner the repopulation of the LPC lesion by astrocytes and remyelination of the axons via OPC recruitment and maturation [12]. Depriving male mice of their testosterone, pharmacological inhibition of CXCR4 with AMD3100, and the conditional genetic ablation of either CXCR4 or AR in astrocytes all completely blocked the formation of new myelin by oligodendrocytes [12]. This was a significant finding, as CXCL12/CXCR4 signaling has shown to induce OPC proliferation, migration, and differentiation into myelin-forming oligodendrocytes [16,17].

However, in the absence of cooperative testosterone and CXCR4 signaling, axons did not remain demyelinated. They were instead remyelinated by Schwann cells invading the lesion [12]. Schwann-cell invasion into astrocyte-poor demyelinating lesions had been already reported in several studies [13,18,19]. These cells may originate from peripheral nerve roots, blood vessels, or progenitor cells. Schwann-cell remyelination has also been observed in MS lesions, mainly in those with a low density of astrocytes that are located within the spinal cord, close to peripheral nerve roots [20,21].

The dependence of myelin repair on the presence of testes and testosterone in males raises the question of the role of ovarian hormones in females. We have previously shown that treating female mice with male-like levels of testosterone promotes remyelination [12,13,22]. Thus, females are sensitive to the remyelinating actions of testosterone. However, levels of endogenous testosterone are an order of magnitude lower in women than in men (0.5–1 nM), as determined by mass spectrometric analysis, and the ovaries mainly produce estradiol and progesterone [23,24,25]. The aim of the present study was to investigate the role of the ovaries and estradiol in myelin repair in female mice. We show that in the absence of ovaries, LPC lesions are not remyelinated by oligodendrocytes and are occupied by Schwann cells instead of astrocytes and oligodendrocytes, as previously observed in castrated male mice. Treatment of ovariectomized female mice with a physiological concentration of estradiol restored the appearance of astrocytes and remyelination by oligodendrocytes within the demyelinating lesion and also opposed the incursion of Schwann cells. However, unlike the remyelinating activity of testosterone, the remyelinating activity of estradiol did not involve cooperation with CXCR4.

## 2. Results

### 2.1. Spontaneous Remyelination in Female Mice and the Effect of Ovariectomy

We used an experimental model in which a focal demyelinating lesion induced by the infusion of lysolecithin (LPC) into the ventral spinal cords of male mice is spontaneously remyelinated by newly formed oligodendrocytes. This endogenous capacity for myelin repair is dependent on the presence of testes or testosterone replacement in castrated male mice [12,13]. The dependence of myelin repair on the presence of testes and testosterone in males raised the question of the role of the ovaries and estradiol in female mice.

**Figure 1:**
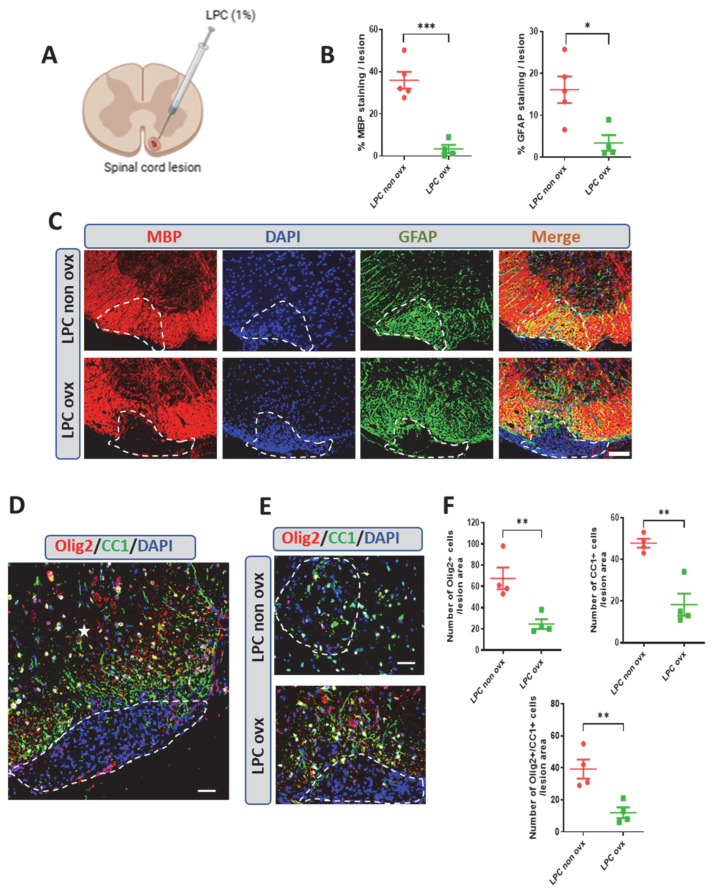
Spontaneous remyelination after LPC-induced demyelination in female mice and the effect of ovariectomy. (**A**) Schematic illustration of LPC lesion in the white matter in the spinal cords of female mice. (**B**,**C**) Quantification of double labeling of myelin (myelin basic protein, MBP) and astrocytes (GFAP) on spinal cord cross-sections from non-ovariectomized (non ovx) and ovariectomized (ovx) female mice at 30 dpl. Cell nuclei were counterstained in blue with DAPI. Within the LPC lesion, which is delimited by a dotted line, strong MBP and GFAP immunostaining colocalized in gonadally intact female mice, whereas MBP and GFAP immunostaining were sparse in ovx female mice. (**D**) Photomicrograph showing immunostaining of Olig2^+^ oligodendroglial cells and CC1+ mature oligodendrocytes in the LPC lesion of a mouse spinal cord, which is delimited by a dotted line, and in the intact area, as indicated by a white star. (**E**,**F**) Quantification of Olig2^+^ oligodendroglial cells and of CC1^+^ mature oligodendrocyte immunostaining on spinal cord cross-sections from non-ovx and ovx female mice at 30 dpl. Within the LPC lesion, which is delimited by the dotted line, both Olig2^+^ and CC1^+^ cells were abundant in non-ovx female mice and sparse in ovx female mice. Most of the Olig2^+^ cells were mature oligodendrocytes coexpressing CC1 at 30 dpl. Data are presented as means ± S.E.M. * *p* < 0.05; ** *p* < 0.01; *** *p* < 0.001 by two-tailed unpaired Student’s *t*-test. Number of animals (*n* = 4–5). Scale bar: 50 µm in (**A**,**C**,**D**) and 20 µm in (**E**).

Here, we show that in gonadally intact female mice, like in male mice, the LPC lesion is completely remyelinated at 30 days post-lesion (dpl), as demonstrated by the recovery of myelin basic protein (MBP) immunostaining. As previously reported for gonadally intact male mice or castrated male mice treated with testosterone, remyelination in female mice was accompanied by the appearance of GFAP^+^ astrocytes, which play an important role in myelin repair (Figure 1B,C).

Just as remyelination in males depends on the presence of testicles, myelin formation in females relies on the presence of ovaries. Indeed, no spontaneous recovery of MBP^+^ myelin or GFAP^+^ astrocytes were observed within the LPC lesions of ovariectomized female mice as late as 30 dpl (Figure 1B,C).

The recovery of MBP-immunoreactive CNS myelin within a demyelinated lesion predominantly requires the recruitment of oligodendrocyte progenitors (OPCs) and their differentiation into myelinating OLs. In female mice with their ovaries, remyelination was indeed accompanied by the replenishment of Olig2^+^ oligodendroglial cells and CC1^+^ mature oligodendrocytes. In contrast, only very few Olig2^+^-, CC1^+^- and CC1-coexpressing Olig2 (Olig2/CC1^+^) cells were observed within the lesion at 30 dpl in the absence of ovaries (Figure 1E,F). Thus, spontaneous myelin repair in the mouse spinal cord is entirely dependent on the presence of ovaries in females, as it is on the presence of testes in males.

### 2.2. Treatment of Ovariectomized Females with Estradiol or Testosterone Restores Remyelination

We previously showed that testosterone treatment with a subcutaneous Silastic implant producing circulating levels of the hormone characteristic of reproductive male mice (10–15 nM) restores myelin repair in both gonadectomized male mice and female mice. Thus, females are sensitive to the remyelinating actions of elevated levels of testosterone [13,22]. However, levels of endogenous testosterone are low in females, and the ovaries mainly produce estradiol and progesterone. We thus examined whether a physiological level of estradiol (0.5–1 nM), delivered by a subcutaneous Silastic implant, restored myelin repair in ovariectomized female mice.

**Figure 2:**
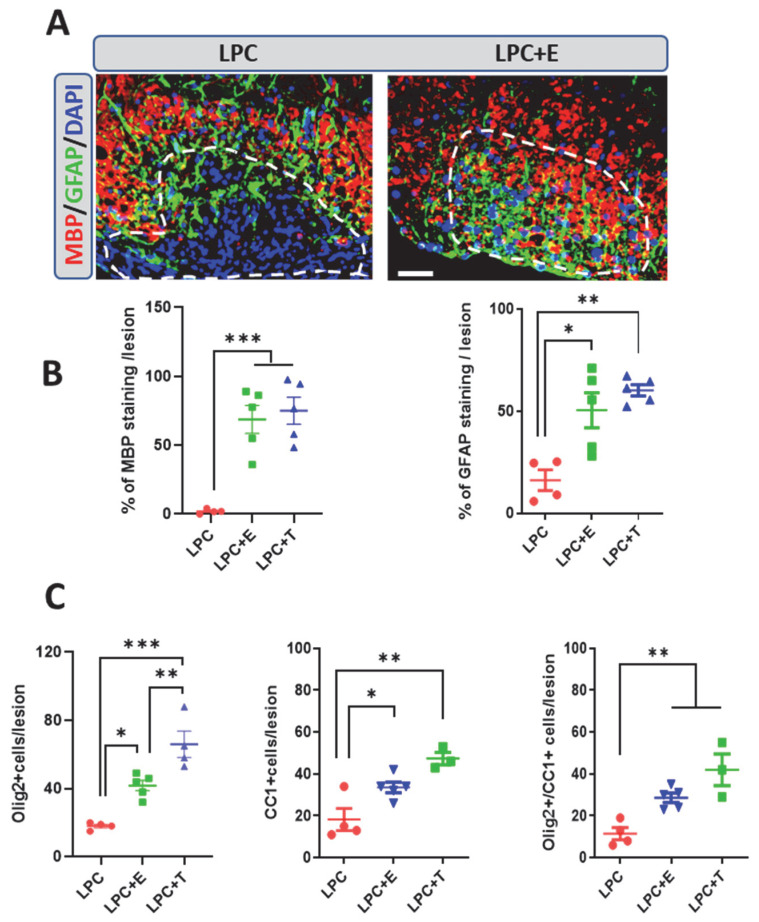
Treatment of ovariectomized female mice with estradiol or testosterone restores remyelination. (**A**) Double labeling of MBP^+^ myelin and GFAP^+^ astrocytes in the LPC-demyelinated lesion on spinal cord cross-sections from ovariectomized female mice that received an empty Silastic implant (LPC) or an estradiol-filled implant (LPC + E) at 30 dpl. Cell nuclei were counterstained in blue with DAPI. Within the LPC lesion, which is delimited by a dotted line, strong MBP and GFAP immunostaining colocalized in the estradiol-treated female mice, whereas both stainings were sparse in female mice that had received an empty implant. (**B**) Quantification of MBP and GFAP immunostaining in female mice that had received an empty implant (LPC), an implant filled with estradiol (LPC + E), or an implant filled with testosterone (LPC + T). (**C**) Counting of Olig2^+^ oligodendroglial cells and CC1^+^ mature oligodendrocytes, as well as the percentage of Olig2^+^ cells expressing the maturation marker CC1 at 30 dpl. LPC: ovariectomized (ovx) female mice that had received an empty implant; LPC + E: ovx female mice that had received an estradiol implant; LPC + T: ovx female mice that had received a testosterone implant. Data are presented as means ± S.E.M. * *p* < 0.05; ** *p* < 0.01; *** *p* < 0.001 by one-way ANOVA with Tukey’s multiple-comparisons tests. Number of animals (*n* = 3–5). Scale bar: 50 µm.

In ovariectomized female mice, treatment with estradiol restored remyelination at 30 dpl, and strong MBP and GFAP staining was observed within the LPC lesion (Figure 2A,B). Thus, as in castrated male mice treated with testosterone, myelin repair accompanied the appearance of astrocytes in the lesion [12]. Results further showed that levels of estradiol typical of female mice restored myelin and astrocytes within the lesion as efficiently as male-like levels of testosterone (Figure 2A,B).

As expected, myelin formation, as measured by the MBP^+^ area, was accompanied by the replenishment of the lesion with Olig2^+^ oligodendroglial cells and CC1^+^ mature oligodendrocytes (Figure 2C). At the doses tested in this experiment, estradiol was slightly but significantly less efficient than testosterone in increasing the number of Olig2^+^ cells, whereas the number of Olig2^+^ cells expressing the CC1 marker did not differ between treatments.

### 2.3. Inhibition of CXCR4 Blocks Testosterone-Dependent Remyelination—But Not Estradiol-Dependent Remyelination

We previously demonstrated cooperation between testosterone and CXCR4 in oligodendrocyte replenishment and remyelination of an LPC-demyelinated lesion. Moreover, testosterone was shown to induce CXCR4 expression within astrocytes. Pharmacological inhibition or conditional genetic ablation of CXCR4 in astrocytes blocked the restoration of MBP^+^ myelin and GFAP^+^ astrocytes by testosterone [12].

**Figure 3:**
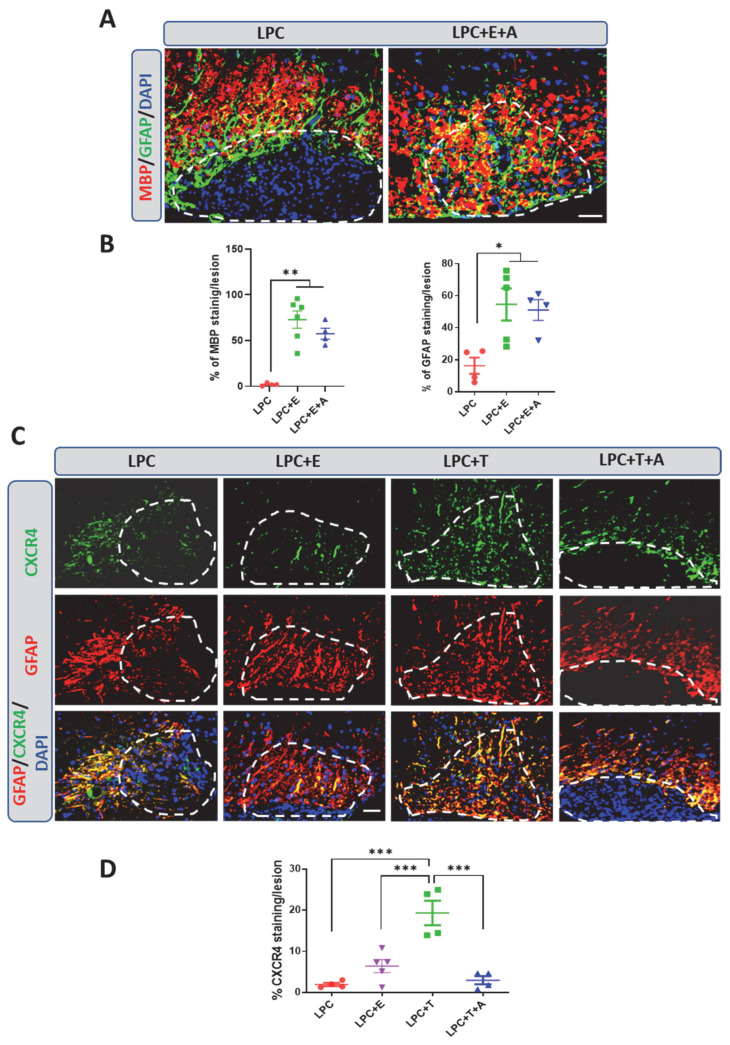
Inhibition of CXCR4 blocks testosterone-dependent remyelination but not estradiol-dependent remyelination in female mice. (**A**,**B**) Double labeling of MBP and GFAP on spinal cord cross-sections from ovariectomized female mice at 30 dpl. Cell nuclei were counterstained in blue with DAPI. Within the LPC lesion, which is delimited by the dotted line, both MBP and GFAP staining were nearly absent in female mice that had received an empty implant (LPC), but MBP and GFAP co-occurred in ovariectomized female mice that had received an implant filled with estradiol (LPC + E). AMD3100, a specific blocker of the CXCR4 receptor, did not inhibit the estradiol-dependent colocalization of MBP and GFAP inside the lesion (LPC + E + A). (**C**,**D**) Double labeling of CXCR4 and GFAP on spinal cord cross-sections from ovariectomized females at 30 dpl. Cell nuclei were counterstained in blue with DAPI. Within the LPC lesion, which is delimited by the dotted line, CXCR4 and GFAP staining were very low in female mice that had received an empty implant (LPC). Only faint CXCR4 immunostaining was observed in astrocytes from female mice treated with estradiol (LPC + E), and this was not significantly different from that observed in the absence of hormone treatment. On the other hand, CXCR4 strongly colocalized with GFAP in female mice treated with testosterone (LPC + T). In this group, AMD3100 blocked the appearance of GFAP^+^/CXCR4^+^ astrocytes within the LPC lesion (LPC + T + A). Data are presented as means ± S.E.M. * *p* < 0.05; ** *p* < 0.01; *** *p* < 0.001 by one-way ANOVA with Tukey’s multiple-comparisons tests. Number of animals (*n* = 4–6). Scale bars: 50 µm.

To assess whether the remyelinating effects of estradiol also involve CXCR4 signaling, ovariectomized female mice that had received an estradiol implant were injected subcutaneously every second day with the selective CXCR4 inhibitor AMD3100 at the previously tested optimal dose of 1 mg/kg [12]. As in the previous experiments, spinal cord sections were examined by immunocytochemistry at 30 dpl, and no MBP^+^ myelin or GFAP^+^ astrocytes were observed within the LPC lesion in ovariectomized female mice that had received an empty implant (Figure 3A). Of note, as already previously observed in male mice [12], astrocytes densely localized around the lesion in the absence of hormone treatment. In response to estradiol, astrocytes filled the lesion and there was a concomitant reduction in the dense GFAP immunostaining at its borders (Figure 3A). In contrast to observations in castrated male mice treated with testosterone, AMD3100 failed to inhibit the replenishment of the lesion with MBP+ myelin and GFAP+ astrocytes in ovariectomized female mice treated with estradiol (Figure 3A,B).

This unexpected result could be interpreted as a fundamental difference in remyelination mechanisms between males and females or as a difference between the remyelinating actions of estradiol and testosterone. To gain insight into the differences between the two hormones, ovariectomized female mice received a Silastic implant that was empty or filled with either estradiol or testosterone. A group of testosterone-treated female mice were concomitantly treated with AMD3100. Lesions were again examined at 30 days after LPC injection for GFAP and CXCR4 colabeling. Both estradiol and testosterone stimulated filling of the lesion with astrocytes. However, whereas astrocytes in the testosterone-treated female mice expressed CXCR4, only faint CXCR4 immunostaining was observed in the estradiol-treated female mice (Figure 3C,D). In line with this observation, AMD3100 blocked the testosterone-dependent appearance of astrocytes within the lesion (Figure 3D). Taken together, these results show that testosterone and estradiol both stimulate remyelination and the appearance of astrocytes within the demyelinated lesion, but do so, respectively, in a CXCR4-dependent or -independent manner.

### 2.4. Estradiol Increases the Number of Oligodendrocytes and Promotes Remyelination in Organotypic Cultures in a CXCR4-Independent Manner

Additional evidence that estradiol stimulates remyelination in a CXCR4-independent manner, whereas inhibition of CXCR4 blocks testosterone-dependent remyelination, was obtained in slice cultures prepared from P10 mouse cerebellum. These organotypic cultures, which preserve the structural and connective organization of neural cells, are a model of choice for neuropharmacological investigations and for studying the myelination of axons [12,22,26,27].

We cultured cerebellar slices from PLP-eGFP mice, in which oligodendroglial cells express eGFP under the PLP gene promoter [28], at postnatal day 10. The slices were partially demyelinated with LPC and treated for 5 days with optimal concentrations of either vehicle (0.1% ethanol), testosterone (0.5 µM), estradiol (1 µM), or either hormone in combination with AMD3100 (5 µM). Results were compared to non-demyelinated control slices.

**Figure 4:**
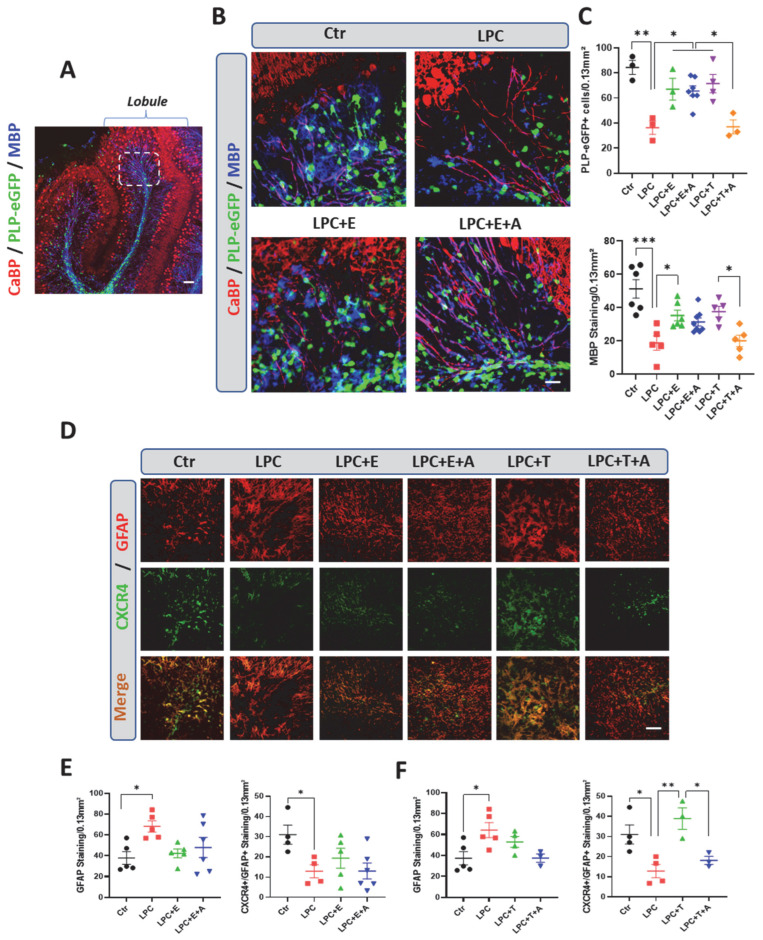
In organotypic cerebellar-slice cultures, inhibition of CXCR4 blocks testosterone-dependent remyelination, but not estradiol-dependent remyelination. (**A**,**B**) Organotypic cultures of sagittal cerebellar slices from postnatal 10 (P10) PLP-eGFP mice. They were triple-stained for eGFP (oligodendroglial cells), MBP (CNS myelin), and calbindin (CaBP, Purkinje neurons). (**A**) Low magnification showing cerebellar lobule and area used for immunostaining quantification (delimited by a dotted line). Control (Ctr) slices were exposed only to vehicle (0.1% ethanol). Treated slices were exposed to 0.5 mg/mL LPC for 18–20 h to cause their demyelination. They were then treated with vehicle, testosterone (T, 0.5 µM); estradiol (E, 1 µM), T + A (AMD3100, 5 µM) or E + A over 5 days. The density of MBP immunostaining and the number of eGFP^+^ oligodendroglial cells were quantified. (**C**) Slices treated with vehicle remained largely demyelinated and depleted of eGFP^+^ oligodendroglial cells compared to controls. Testosterone or estradiol treatment restored MBP^+^ myelin and replenished eGFP^+^ cells. The effects of testosterone, but not of estradiol, were inhibited by AMD3100 (respectively, LPC + T + A and LPC + E + A). (**D**–**F**) Organotypic cultures of sagittal cerebellar slices from P10 mice were used for analyzing CXCR4 and GFAP immunostaining and their colocalization after treatment with testosterone (T) or estradiol (E, F). Exposure to LPC significantly increased numbers of reactive GFAP^+^ astrocytes and decreased expression of CXCR4 in treated slices compared to control (Ctr) slices. GFAP^+^ astrocytes did not significantly increase in response to testosterone or estradiol, and CXCR4 increased significantly only in slices treated with testosterone, an effect that was blocked by AMD3100 (A). Estradiol did not affect GFAP or CXCR4 staining compared to levels in LPC slices treated with vehicle. Data are presented as means ± S.E.M. * *p* < 0.05; ** *p* < 0.01; *** *p* < 0.001 by one-way ANOVA with Tukey’s multiple-comparisons tests. Number of animals (*n* = 3–7). Scale bars: 20 µm.

LPC-demyelinated slices treated with vehicle were significantly depleted of eGFP^+^ oligodendroglial cells, and MBP-immunoreactive myelin was sparse in these slices compared to the controls (Figure 4B,C). Treatment with estradiol or testosterone restored the density of eGFP^+^ oligodendroglial cells and of MBP^+^ myelin to levels comparable to those seen in the controls. The preserved cellular structures in these cultures revealed that eGFP^+^ oligodendroglial cells and MBP^+^ myelin were aligned along CaBP^+^ Purkinje cell axons in the control and hormone-treated slices. As occurs in vivo, replenishment with oligodendroglial cells and remyelination by testosterone, but not by estradiol, were blocked by treatment with AMD3100 (Figure 4B,C).

GFAP immunostaining and its colocalization with CXCR4 staining were then examined in cerebellar slice cultures prepared from wild-type mouse pups. In these cultures, GFAP immunostaining was increased and CXCR4 was decreased in response to LPC exposure in treated slices compared to control slices (Figure 4D–F). However, there was minimal colocalization between GFAP and CXCR4 staining in the absence of hormone treatment, indicating that the LPC-treatment reduces CXCR4 expression by astrocytes. Both estradiol and testosterone restored GFAP staining to control levels. However, whereas testosterone upregulated CXCR4 expression within astrocytes, CRXCR4 staining remained sparse in the estradiol-treated slices. Importantly, AMD3100 inhibited the increase in CXCR4 otherwise induced by testosterone but had no effect on the weak CXCR4 immunostaining observed in estradiol-treated slices (Figure 4D–F). Taken together, these results confirm the cooperative role of testosterone and CXCR4 in promoting remyelination. They also show that testosterone induces CXCR4 expression in astrocytes; this effect must require concerted signaling by testosterone and CXCR4, as it could be inhibited by treatment with AMD3100. In contrast to testosterone, estradiol stimulates both the appearance of astrocytes and remyelination within the lesion independently of CXCR4. Moreover, estradiol does not induce CXCR4 expression in astrocytes.

### 2.5. In the Absence of Estradiol and Oligodendrocyte Remyelination, Schwann Cells Invade and Remyelinate the Demyelinated Lesion

Astrocytes expressing a pro-regenerative phenotype provide support for remyelination by oligodendrocytes [29,30]. Moreover, within the spinal cord, axons are remyelinated by Schwann cells derived from the peripheral nervous system (PNS) in the absence of astrocytes [31]. Also, the paucity of astrocytes resulting from testosterone deficiency or the absence of a functional androgen receptor in male mice lead to extensive Schwann-cell-dependent remyelination. By contrast, testosterone-dependent remyelination by oligodendrocytes prevents the invasion of Schwann cells into the LPC-induced lesion [12,13].

**Figure 5:**
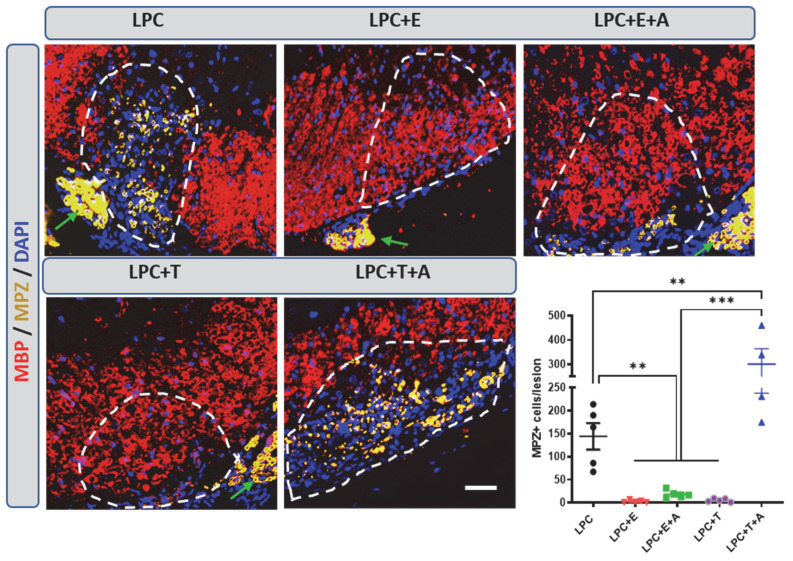
Migration of Schwann cells into an LPC-demyelinated area is prevented by estradiol in a CXCR4-independent manner. Double labeling of peripheral myelin and Schwann cells (MPZ) as well as of CNS myelin (MBP) on spinal cord cross-sections from ovariectomized female mice at 30 dpl. Cell nuclei were counterstained in blue with DAPI. Within the LPC lesion, which is delimited by a dotted line, MPZ immunostaining was observed in female mice that had received an empty Silastic implant (LPC). In female mice treated with estradiol (LPC + E) or testosterone (LPC + T), the lesion was entirely occupied by MBP^+^ myelin, excluding MPZ immunostaining. The restoration of MBP^+^ myelin by testosterone was inhibited by AMD3100; the restoration of MBP^+^ myelin by estradiol was not (A). As a consequence, oligodendrocyte remyelination was replaced by Schwann-cell-dependent remyelination (MPZ^+^) in female mice treated with testosterone + AMD3100 (LPC + T + A) but not in female mice treated with estradiol + AMD3100 (LPC + E + A). Schwann cells appear to originate from peripheral nerve roots (indicated by green arrows). Data are presented as means ± S.E.M. ** *p* < 0.01; *** *p* < 0.001 by one-way ANOVA with Tukey’s multiple-comparisons tests. Number of animals (*n* = 4–5). Scale bars: 20 µm.

To investigate whether in the absence of gonadal hormones, axons are also remyelinated in female mice by Schwann cells, ovariectomized female mice were treated or not treated with estradiol or testosterone after LPC demyelination. In ovariectomized female mice treated with vehicle, MBP^+^ CNS myelin remained sparse at 30 dpl, whereas abundant myelin protein zero (MPZ) immunostaining, reflecting Schwann-cell-dependent remyelination, was observed within the lesion (Figure 5). Both estradiol and testosterone restored MBP^+^ CNS myelin and opposed the appearance of MPZ^+^ peripheral myelin. Consistent with the CXCR4 dependence of testosterone-induced oligodendrocyte remyelination and the CXCR4 independence of estradiol-induced oligodendrocyte remyelination, AMD3100 blocked the repelling effect of testosterone, but not that of estradiol, on Schwann-cell-dependent remyelination (Figure 5).

## 3. Discussion

Herein, we demonstrate that spontaneous remyelination by oligodendrocytes in the spinal cords of female mice is dependent on the presence of the ovaries and estrogens. This reliance of myelin repair in the spinal cord on functional gonads, the ovaries in females and, as previously shown, the testes in males, is stunning and unveils a key role of estrogens and androgens. Pro-remyelinating actions of estrogens and androgens and favorable effects of the presence of gonads have been previously shown in a variety of experimental systems, but they were not predictive of the strong hormonal dependency observed here [32,33]. One potential explanation for the complete absence of oligodendrocyte-mediated remyelination in the spinal cord following gonadectomy is the rapid infiltration of the lesion site by Schwann cells originating from peripheral nerve roots. This early occupation may create a competitive environment that inhibits differentiation of oligodendrocyte precursor cells (OPCs). Interestingly, while many myelinating Schwann cells in the spinal cord are indeed derived from the peripheral nervous system, studies have shown that a subset also originates from CNS-resident OPCs, underscoring the remarkable plasticity of the glial lineage within the central nervous system [19,34].

Indeed, in castrated mice, Schwann cells rapidly enter the area of demyelination at a time when it is still poor in astrocytes [12]. Astrocytes are also well known to exclude Schwann cells from the CNS [35,36]. Schwann cells have previously been shown to repopulate gliotoxin lesions [31,37], and Schwann-cell-dependent remyelination has been observed in MS lesions, particularly within areas poor in astrocytes. Moreover, Schwann cells are more abundant in spinal-cord plaques, in proximity to peripheral nerves, than in brain lesions [20,38].

Treatment of ovariectomized female mice with a physiological dose of estradiol restored the reappearance of astrocytes within the LPC lesion and remyelination by oligodendrocytes, demonstrating that estradiol therapy is sufficient to restore the regeneration of myelin. Estrogens control transcriptional responses through binding to two different receptors, estrogen receptor alpha (ERα) and beta (ERβ) [39]. Using female transgenic mice with conditional ablation of either ERα or ERβ in different neural cell types, it has been demonstrated that ERα in astrocytes and ERβ in oligodendrocytes mediate neuroprotective and remyelinating effects of estrogens in experimental autoimmune encephalomyelitis (EAE) [40,41,42,43]. Estrogen-receptor signaling in astrocytes supports remyelination by reducing inflammation, increasing levels of neurotrophic factors such as BDNF and IGF-1 [44] and promoting cholesterol biosynthesis critical for myelin formation by oligodendrocytes [45]. Astrocyte-specific deletion of ERα leads to worsened clinical outcomes and axonal damage in EAE models [42], while ERβ activation enhances repair by upregulating lipid metabolism and increasing CXCL1 expression; CXCL1, in turn, binds to CXCR2 receptors on oligodendrocytes [46]. These mechanisms collectively create a permissive environment for oligodendrocyte precursor cell (OPC) survival, recruitment, and differentiation.

More recently, it has been shown that ERβ in astrocytes is also involved in neuroprotection in aging female mice [47]. In addition, both ER subtypes also mediate anti-inflammatory and immunomodulatory effects of estrogens [43,48,49].

Although estradiol therapy was sufficient to restore remyelination after ovariectomy, the hormone is likely to cooperate with progesterone in reproductive females, where it is synthesized by the corpus luteum and the adrenal glands and also synthesized locally within the brain and spinal cord [50,51,52]. Progesterone indeed exerts strong promyelinating effects by acting on intracellular progesterone receptors, as demonstrated in organotypic cultures of brain slices and in vivo after toxin-induced demyelination [27,53,54]. Combined treatment with estradiol and progesterone has indeed been shown to stimulate remyelination more efficiently than their individual administration does [55,56,57].

Estrogen treatment has beneficial effects in EAE and stimulates remyelination in both female and male mice [33,58]. Whereas the ovaries are the principal source of estrogens in females, estradiol is mainly formed from testosterone by the aromatase enzyme in males [44]. While AR signaling is required for testosterone-dependent myelin repair in males, its aromatization to estradiol also plays a role in differentiation of OPC into premyelinating oligodendrocytes [15]. Whereas estradiol is a major remyelinating hormone in females, its acts in a concerted manner with low levels of androgens for proper myelin regeneration [59].

In the spinal cords of female mice, the remyelinating actions of testosterone and estradiol involve different mechanisms. Whereas testosterone-dependent recruitment of astrocytes and remyelination by oligodendrocytes require cooperation between testosterone and CXCR4 signaling, both processes are stimulated by estradiol in a CXCR4-independent manner, as they are not inhibited by the selective CXCR4 antagonist AMD3100. Also, AMD3100 blocked the opposing effects of testosterone, but not of estradiol, on the incursion of Schwann cells into the area of demyelination. Accordingly, testosterone, but not estradiol, induced CXCR4 expression in astrocytes, as was seen after focal LPC-induced demyelination in the spinal cord and in LPC-demyelinated cerebellar slices in culture.

We demonstrate here that the key role of functional ovaries and estradiol in the regeneration of CNS myelin by oligodendrocytes provides further strong preclinical support for estrogen-replacement therapy in women with MS at menopause. The loss of ovarian estrogen production has indeed been associated with increased vulnerability of the brain and deleterious effects on the progression of disability in MS [60,61]. There has been concern about an increased risk of breast cancer and cardiovascular risks associated with menopausal estrogen therapy. However, the type of estrogen, as well as the dose and mode of administration, are important considerations. Treatment of menopausal women with a low dose of transdermal instead of oral estradiol, even over extended periods, may not be associated with breast cancer and shows a neutral effect on cardiovascular risks [62,63,64]. An interesting option is the use of tissue-selective estrogens, but their specific effects on myelin repair need to be evaluated [65]. For the treatment of women with multiple sclerosis, a recent Phase 2 trial has provided evidence for beneficial effects of the pregnancy estrogen estriol in combination with an immunomodulator [66,67]. Benefits of estriol are based on its preference for ERβ, which is involved in neuroprotection and remyelination, whereas ERα also mediates peripheral actions of estrogens [68,69]. Notably, estriol offers an array of health benefits for postmenopausal women [70].

## 4. Materials and Methods

### 4.1. Animals

Mice were housed in standard plastic cages with 1–5 littermates in a 12-h/12-h light/dark cycle in a temperature-controlled room (~21 °C), with ad libitum access to food and water. Approval of the animals’ care and bioethical approval for the in vivo experiments were obtained in accordance with institutional policies and guidelines (INSERM, French and European Community council directive 86/806/EEC). Wild-type and transgenic mice were used at postnatal day 10 (P10) for the preparation of organotypic slice cultures and between 8 and 12 weeks of age for the production of demyelinating spinal cord lesion with lysolecithin (LPC). All mice were bred on the C57BL6/J background.

Female mice were ovariectomized 2–3 weeks prior to the LPC treatment that induced lesion formation. They showed very low levels of testosterone and estradiol. Levels of estradiol were below the detection limit of 100 pM for both sexes [12].

PLP-eGFP mice expressing enhanced green fluorescent protein (eGFP) in cells of the oligodendroglial lineage under the control of the myelin proteolipid protein (PLP) gene promoter were obtained from Wendy Macklin (University of Colorado, Aurora, CO, USA) [28].

### 4.2. Focal Demyelination with Lysolecithin (LPC)

Demyelination was induced by injection of 1% LPC (lysophosphatidylcholine or lysolecithin, Sigma-Aldrich, St. Quentin Fallavier, France) in phosphate-buffered saline (PBS) into the right ventral funiculus of 2–3 months old castrated male or ovariectomized female mice anesthetized with ketamine (80 mg/kg) and xylazine (10 mg/kg). The LPC was injected bilaterally at 0.6 mm lateral to the midline of the spinal cord and at depths of 1.43 to 1.46 mm between T11 and T12. The central vein was used as a benchmark. Injection was performed with glass capillaries (diameters not exceeding 50 µm) connected to a Hamilton syringe controlled by an infusion pump delivering 0.1 µL/min for 10 min. Under these experimental conditions, the staining of large-caliber axons with an antibody against neurofilament 200 kDa (NF-200) revealed no differences after LPC-induced demyelination between animals that had received an empty or a T-filled s.c. implant at 30 days post-lesion (dpl) [12,13]. After LPC infusion, ovariectomized female mice received, at the end of the surgical procedure, a subcutaneous (s.c.) 10 mm Silastic implant (inner diameter: 1.57 mm; outer diameter: 2.41 mm; Dow Corning) filled with testosterone and producing male-like levels of the hormone (10–15 nM) or a 5 mm implant filled with estradiol and producing female-like levels of the hormone (0.5–1 nM), as determined by the GC/MS technique [12,13]. Controls received an empty implant. After dose optimization, AMD3100 (1 mg/kg, Sigma-Aldrich, St. Quentin Fallavier, France) was subcutaneously injected every 2 days [12]. Mice were sacrificed at 30 dpl for immunohistological analysis.

### 4.3. Organotypic Cerebellar Slice Cultures

Cultures of 350 µm thick cerebellar slices were prepared from P10 PLP-eGFP mouse pups, as described earlier [22,71]. Slices were cultured on Millipore membranes 30 mm in diameter with a 0.4 µm pore size (Millicell, Millipore, Fontenay sous Bois, France) at 37 °C in a 5% carbon dioxide (CO_2_) humidified atmosphere. The medium was composed of 50% basal medium with Earle’s salts (Invitrogen, Gaithersburg, MD, USA), 25% Hank’s balanced salt solution (Life Technologies, Villebon Courtaboeux, France), 25% horse serum (Life Technologies), L-glutamine (1 mM) and 5 mg/mL glucose. The medium was changed every 2 to 3 days. To induce demyelination after 7 days in culture (DIV), 0.5 mg/mL LPC (Sigma-Aldrich) was added in fresh medium for 18–20 h. After removing the medium, slices were incubated for 5 additional days in the absence or presence of testosterone or estradiol (0.5 and 1 µM in 0.1% ethanol, respectively). AMD3100 was added at the concentration of 5 µM. These doses had been previously optimized [12]. Five to six slices per cerebellum per animal and at least three animals were used for each treatment.

### 4.4. Immunohistochemistry

Mice were deeply anesthetized with a ketamine–xylazine mixture and then fixed by transcardiac perfusion with a 4% freshly prepared paraformaldehyde solution in PBS (0.1 M, pH 7.5). Extracted spinal cords were post-fixed using 4% paraformaldehyde, embedded in paraffin blocks, and sectioned transversely at 5 µm thickness using a microtome (Micro HM 340E, ThermoScientific, Villebon Courtaboeux, France). After deparaffinization, rehydration, and epitope retrieval in 1× citrate buffer solution, sections were blocked for 1 h in Sea Block Blocking Buffer (Sigma-Aldrich, St. Quentin Fallavier, France) before immunostaining. Primary antibodies (rabbit anti-MBP, Abcam, Paris, France, RRID: AB_92396; mouse anti-MBP, Millipore, RRID: AB_11212910; rat anti-MBP, Millipore, RRID: AB_94975; rabbit anti-Olig2, Millipore, RRID: AB_570666; mouse anti-Adenomatous Polyposis Coli (CC1), Millipore, RRID: AB_2057371; mouse anti-Calbindin D-28K, Swant, Burgdorf, Switzerland, RRID: AB_10000340; Human CXCR4 (4G10), Santa Cruz, Heidelberg Germany, RRID: AB_782002; mouse anti-Glial Fibrillary Acidic Protein (GFAP), Sigma-Aldrich, RRID: AB_11000751; Glial Fibrillary Acidic Protein (multipurpose) antibody, Agilent, Les Ulis, France, RRID: AB_477010; P-Zero Myelin Protein (PZO) antibody (P0), Aves Labs, Davis, CA, USA, RRID: AB_839504) were incubated with the samples overnight at 4 °C; incubation was followed by PBS 1× washing and incubation with secondary antibodies (anti-mouse Alexa488-conjugated, Thermo Fisher Scientific, Villebon Courtaboeux, France, RRID: AB_2535720; anti-mouse Alexa633-conjugated, Thermo Fisher Scientific, RRID: AB_2338006; anti-rat Cy3-conjugated, Jackson Immunoresearch, Cambridgeshire, UK, RRID: AB_2535794) at room temperature for 2 h. Organotypic cultures of cerebellar slices were fixed using PFA 4% to perform immunofluorescence, as previously described [27].

### 4.5. Immunohistological Analysis

Processed spinal cord slices were permanently mounted, and pictures were taken using confocal (Zeiss LSM510-Meta, Voisins-le-Bretonneux, France) and AxioImager A1 microscopes, Oberkochen, Germany). For each cerebellar slice culture, three different cerebellar lobules were photographed. For spinal cord sections, the centers and borders of demyelinated areas (between T11 and T12) were photographed. All analysis was performed with NIH Fiji software (ImageJ 2.1.0/1.54f; NIH, Bethesda, MD, USA). The lesion area was delimited, in LPC lesions, by the dense DAPI staining within the lesion site and the strong GFAP+ staining at the borders. In LPC+E- and LPC+T-treated animals, lesions were delimited by strong expression of GFAP and/or CXCR4 markers in the center of lesion, which showed a marked difference in comparison to the periphery of lesion.

To evaluate the in vivo expression of MBP, GFAP, and CXCR4, immunostaining densities were quantified. An arbitrary threshold was set for the standardization of the different measurements. The quantification of fluorescence was normalized to the lesion area. For all tissues (lesioned and lesioned + testosterone), regions of interest (ROI) were isolated from the rest of the image and areas outside of the ROI were cleared. A threshold was set for the standardization of different measurements, and the same threshold intensity range was maintained for all images. Then, the area covered by a positive staining signal (immunolabelled material: gray scale pixels) was calculated as a percentage of staining within the ROI area by dividing the number of grayscale pixels by the total number of gray- and white-scale pixels multiplied by 100. White-scale pixels correspond to the area covered by the background “noise” (non-specific staining). Markers of oligodendrocytes (Olig2, CC1) and Schwann cells (MPZ) were counted within the LPC lesion area. In organotypic slice cultures, five to six cerebellar slices per animal per insert were used and the experiment were repeated three times. Lesions are multiple and diffuse, so a surface of 0.13mm^2^ was defined in the same lobule for quantification of GFAP and CXCR4 immunolabeling and to count PLP-eGFP^+^ cells.

### 4.6. Statistical Analysis

G*Power 3.1.9.2 software was used to calculate the required sample size. The minimal significance (α) and statistical power (1 − β) were set at 0.05 and 0.7, respectively, to detect a difference of 30%. Group sizes were calculated based on our previous studies. Differences between multiple groups were analyzed by one-way ANOVA followed by Tukey’s multiple-comparisons tests. Differences between two groups were analyzed by two-tailed unpaired Student’s *t*-tests (GraphPad Prism, v.8 GraphPad Software, La Jolla, CA, USA). Data from three separate experiments are presented as means ± S.E.M, and significant differences are marked by asterisks (*** *p* < 0.001, ** *p* < 0.01, * *p* < 0.05). The experimenter/analyzer was blinded to experimental conditions.

## Data Availability

The original contributions presented in this study are included in the article. Further inquiries can be directed to the corresponding author.

## Abbreviations

GFAP = glial fibrillary acidic protein; OPC= oligodendrocyte progenitor cells; MS = multiple sclerosis; T = testosterone; E = estradiol; EAE = experimental autoimmune encephalomyelitis; LPC = lysolecithin; MBP = myelin basic protein; MPZ = myelin protein zero; PLP = myelin proteolipid protein; eGFP = enhanced green fluorescent protein; Olig2 = oligodendrocyte lineage transcription factor 2; CC1 = mature oligodendrocyte marker; DAPI = 4′,6-diamidino-2-phénylindole.
